# The Longitudinal Associations between Perceived Descriptive Peer Norms and Eating and Drinking Behavior: An Initial Examination in Young Adults

**DOI:** 10.3389/fpsyg.2017.00002

**Published:** 2017-01-23

**Authors:** Andrew Jones, Eric Robinson

**Affiliations:** Psychological Sciences, University of LiverpoolLiverpool, UK

**Keywords:** eating norms, descriptive norms, social eating, social norms

## Abstract

Experimental and cross-sectional studies indicate that perceptions of the eating and drinking behavior of one's peers (perceived descriptive peer norms) are associated with the types, frequency and quantity of food, and beverages a person chooses to consume. At present, we know very little about the longitudinal association between perceived descriptive peer norms and future eating or drinking behavior. In this study, we examined whether perceived descriptive peer norms for different food/beverage types predicted frequency of consumption of food/beverages in university students. Three hundred and forty participants completed measures at baseline and follow-up for frequency of consumption of cakes/pastries, sugar containing beverages, and alcoholic beverages, as well as measures of perceived descriptive peer norms at both time points. Perceived descriptive peer norms predicted consumption of pastries/cakes at follow up when controlling for changes in these perceptions over time; believing that one's peers frequently consumed cakes/pastries was associated with an increased frequency of consumption over time, although the magnitude of this effect was small. There was no significant association between perceived descriptive peer norms and changes in frequency of consumption of sugar containing beverages or alcohol over time. In the present longitudinal study of young adults, beliefs about how often one's peers eat or drink specific food and beverages types had limited effect on future eating and drinking behavior.

## Introduction

Perceived descriptive peer norms refer to beliefs about how a person's peers behave. For example, a person could correctly or incorrectly believe that it is common for their peers to frequently drink sugar sweetened beverages. Although the influence perceived descriptive peer norms have on behavior has been of interest to social and health psychologists for some time (Cialdini et al., 1990; Terry et al., 1999), recently there has been an increasing awareness of their potential relevance to eating and drinking behavior (Robinson et al., 2013a; Higgs, 2015). Descriptive peer norms are thought to influence behavior through informational social influence; “if everyone else is doing it, then maybe it would be wise for me to do also” (Deutsch and Gerard, 1955). In a laboratory setting, there is now convincing evidence that perceived descriptive peer norms can influence consumer behavior (Robinson et al., 2014c). For example, learning that others in a laboratory study have been eating a lot of food typically results in participants increasing their food intake (Prinsen et al., 2013; Robinson and Field, 2015). Likewise, it has been shown that if children are led to believe that their peers are eating a lot of vegetables they increase their own consumption of vegetables (Sharps and Robinson, 2015, 2016). A smaller number of intervention studies have examined whether providing consumers with information about descriptive peer norms influences future consumer behavior measured immediately afterwards or up to 1 week later, but results have been mixed (Stok et al., 2012; Mollen et al., 2013; Robinson et al., 2013b, 2014a; de Bruijn et al., 2015; Verkooijen et al., 2015). Thus, at present the long term effect that perceived descriptive peer norms have on behavior is unclear (Robinson, 2015).

Outside of the laboratory there are now a number of cross-sectional studies that have examined the relationship between perceived descriptive peer norms and consumer behavior. In particular, it has been shown that believing one's peers frequently eat or drink a food or beverage type correlates with how often a person consumes those foods/beverages (Louis et al., 2007; Ball et al., 2010; Lally et al., 2011; Robinson et al., 2014b, 2016; Staunton et al., 2014; Pelletier et al., 2016). One interpretation of these findings is that perceived descriptive peer norms influence consumer behavior by informing consumers what an appropriate way to behave is (Robinson, 2015). However, studies examining only the cross-sectional association between perceived descriptive peer norms and consumer behavior do not provide convincing evidence for the causal influence of perceived descriptive peer norms. This is because reverse causality could in part explain the association between personal consumption frequency and perceived descriptive peer norms; we often base our beliefs about how our peers behave on our own behavior, otherwise known as the false consensus effect (Marks and Miller, 1987). Moreover, few studies have examined whether there is a longitudinal association between perceived descriptive peer norms and consumption behavior (Robinson, 2015). In a rare exception, Louis and colleagues conducted a small study examining eating behavior and perceived descriptive norms over the course of 1 week and found no evidence that perceived descriptive norms predicted behavior 1 week later (Louis et al., 2007). Thus, at present it is not clear whether believing that one's peers regularly eat a food increases the likelihood that a person will more frequently consume that food in the future. Nor is it clear whether changes to perceived descriptive peer norms over time result in changes to eating or drinking behavior.

The aim of the present study was to examine whether perceived descriptive norms relating to the frequency by which others consume different food/ beverage types predict the frequency by which these food types are consumed a year later in a cohort of UK university students. To test this we examined whether baseline perceived descriptive norms and/or changes in perceived descriptive norms over time predicted future personal consumption of different food/beverage items. Using the present cohort study, we have previously examined cross-sectional associations between perceived descriptive peer norms and personal consumption frequency of cakes/pastries, sugar sweetened beverages, and alcohol. Results indicated that believing one's peers frequently consume these food/beverage types was associated with a higher personal frequency of consumption (Robinson et al., 2014b, 2016). We also observed that trait self-control moderated this relationship; individuals with low self-control were more likely to eat/drink in line with perceived descriptive norms (Robinson et al., 2014b, 2016). We speculated that this effect may have been explained by individuals with low self-control being less able to inhibit normative influences on their eating/drinking behavior (Burkley et al., 2011; Salmon et al., 2014). Thus, in the present study we also examined whether trait self-control moderated any longitudinal associations between perceived descriptive norms and frequency of food/beverage consumption. Based on the results of previous cross-sectional analyses (Robinson et al., 2014b, 2016) we predicted that perceived descriptive norms would predict future consumption behavior and that these effects may be particularly pronounced among individuals with low levels of trait self-control.

## Methods

### Participants

Participants were part of a cohort of university students (University of Liverpool, UK), recruited into a study designed to assess precursors and antecedents of health behaviors. Aside from being a University of Liverpool student there were no further inclusion or exclusion criteria for participation. Students were recruited through mailshots and intranet advertisements, and participated in exchange for entry into a small monetary prize draw. Data was collected via the internet using survey administration software and the study was approved by the University of Liverpool Research Ethics Committee.

Data collection at baseline took place during February 2014 and follow up was during February 2015. At baseline we collected data from 1056 (307/29% male) students, with an average age of 21.7 (±4.5) years. All participants who participated at baseline were contacted 1 year later. At follow up, 410 individuals attempted the survey with 340 completing all questionnaires (89/26% male). Therefore, our final retention rate was 32%. We collected data on numerous variables and the full set of measures included at baseline is described elsewhere (Robinson et al., 2014b, 2016). Measures were completed in a randomized order and the questionnaire battery in each wave took ~10–15 min to complete. Wave one consisted of 115 and wave two consisted of 136 individual responses to questions or statements.

For the present study, we analyzed data on frequency of consumption of alcohol, cakes/pastries and sugar containing sodas, as these were the only food/beverage items we also measured perceived descriptive peer norms for. We also made use of measures of trait self-control, Body Mass Index, age, and gender in our analyses.

### Measures

#### Demographics

Demographic variables included age, gender, and BMI (calculated using self-reported height and weight) at baseline. Self-control was measured using the Brief Self-Control Scale (BSCS: Tangney et al., 2004), also assessed at baseline. The measure consists of 13 items (e.g., “I usually think carefully before doing anything”) and has good internal consistency and reliability.

#### Frequency of food/beverage consumption

To examine individual's consumption of each food/beverage item at both time points we used a food frequency questionnaire. This contained items relating to frequency of consumption for both cakes/pastries and sugar containing soda using similar statements (e.g., “*Please indicate how often you eat […]*”) with possible scored answers; never (0), less than yearly (1), once a year (2), several times per year (3), once per month (4), 2–3 times per month (5), once per week (6), 2–6 times per week (7), or daily (8). Frequency of heavy alcohol consumption was measured using item 3 of the Alcohol Use Disorders Identification task “*How often do you have six or more drinks on one occasion?*,” with possible answers including; never (0), less than monthly (1), monthly (2), weekly (3), daily or almost daily (4). We measured frequency of heavy alcohol use (as opposed to frequency of any alcohol use) because frequent hazardous drinking is common among student populations and is linked to substantial health and behavioral consequences (Wechsler et al., 1994; Stephens and Duka, 2008).

#### Perceptions of descriptive peer norms

As in previous studies (Robinson et al., 2014b, 2016) perceptions of descriptive peer norms were assessed at both time points using single statements with five possible responses. To assess frequency of cakes/pastry, sugar containing soda and alcohol consumption we asked “*How often do you think most students eat/drink […]?*” with possible scored answers including never (0), less than monthly (1), monthly (2), weekly (3), daily, or almost daily (4).

### Analysis strategy

We conducted paired-samples *t*-tests to examine whether there were differences between time point 1 and time point 2 for frequency of consumption and/or perception of norms of the individual food/drink items. To assess the effects of perceived descriptive norms on future consumption behavior, we conducted block adjusted multiple linear regression analyses, separately for each food/beverage type. The dependent variable in each regression was the frequency of consumption of each food or drink type during time 2. In the first block, we entered the demographic variables; age, gender, and BMI, along with frequency of consumption at baseline to statistically control for their influence. In block two, we entered perceptions of descriptive peer norms at baseline, in block three, we entered the change in perceptions of peer norms from baseline to follow up, along with trait self-control scores. In block four, we entered interaction terms between trait self-control and perceptions of peer norms at baseline, as well as between trait self-control and change in perceptions in peer norms from baseline to follow up.

## Results

### Change in frequency of consumption and perceptions of norms over time (Table 1)

There was evidence of variability in changes to personal consumption of the different food/beverage types from baseline to follow up. For cake/pastries, 31.9% of participants had the same consumption frequency from baseline to follow up, with 36.6% decreasing and 31.5% increasing frequency of consumption. For sugar containing sodas, 35.1% of participants had the same consumption frequency from baseline to follow up, whilst 39.5% decreased and 25.4% increased their frequency of consumption. For alcohol, 62.2% of participants had the same consumption frequency from baseline to follow up, with 24.9% decreasing and 12.9% increasing frequency of consumption.

In the overall sample there was a significant decrease in the number of times individuals drank six or more drinks on one occasion [*t*_(339)_ = 3.41, *p* < 0.001], and in consumption of sugar containing sodas [*t*_(339)_ = 3.05, *p* = 0.002] from baseline to follow up. Change in the consumption frequency of cakes/pastries from baseline to follow up was not significant [*t*_(339)_ = 1.28, *p* = 0.20]. Perceptions of descriptive peer norms did not change for alcohol [*t*_(339)_ = 0.65, *p* = 0.52] or cake/pastry consumption [*t*_(339)_ = 0.88, *p* = 0.381]. Perceptions of peer consumption of sugar containing soda reduced significantly from baseline to follow up [*t*_(339)_ = 5.08, *p* < 0.001]. See Table 1.

**Table 1:** **Changes in consumption and norm perceptions from baseline to follow up**.

	**Time 1**		**Time 2**	
	**Mean**	***SD***	**Mean**	***SD***
**FREQUENCY**				
Cakes/pastries	4.99	1.49	4.89	1.50
Sugar containing soda	4.01	2.35	3.69	2.35
Six or more alcoholic drinks	1.52	0.98	1.38	0.92
**PERCEPTIONS OF NORMS**				
Cakes/pastries	2.96	0.69	2.99	0.62
Sugar containing soda	3.63	0.54	3.43	0.66
Six or more alcoholic drinks	2.70	0.55	2.73	0.53

*Cakes/pastries and sugar containing soda: minimum score 0, maximum score 8. Six or more alcoholic drinks: minimum score 0, maximum score 4. Higher scores indicating greater consumption frequency*.

We report correlations between frequency of consumption and peer norms for cakes/pastries, sugar containing soda, and alcohol at each time point in Table 2. To briefly summarize, we note modest correlations between peer norms and frequency of consumption of cakes/pastries and alcohol but not sugar containing soda, across both time points.

**Table 2:** **Correlations between variables across each time point**.

	**1**	**2**	**3**	**4**	**5**	**6**	**7**	**8**	**9**	**10**	**11**	**12**
1. Cons. Cakes/Pastries (T1)	–	0.18^**^	−0.17^**^	0.54^**^	0.09	−0.16^*^	0.37^**^	0.14^**^	−0.04	0.22^**^	−0.03	−0.04
2. Cons. Soda (T1)		–	0.15^**^	0.16^**^	0.66^**^	0.08	0.05	0.08^*^	0.16^**^	0.15^**^	0.08	0.10
3. Cons. Alcohol (T1)			–	0.02	0.23^**^	0.71^**^	−0.03^**^	−0.07	0.39^**^	0.00	0.013	0.34^**^
4. Cons. Cakes/Pastries (T2)				–	0.24^**^	0.00	0.27^**^	0.09	0.00	0.29^**^	0.00	−0.01
5. Cons. Soda (T2)					–	0.14^*^	−0.02	0.05	0.16^**^	0.07	0.09	0.07
6. Cons. Alcohol (T2)						–	−0.06	−0.10	0.28^**^	−0.02	−0.05	0.29^**^
7. Peer norms Cakes/Pastries (T1)							–	0.38^**^	0.12^*^	0.47^**^	0.21^**^	0.07
8. Peer norms Soda (T1)								–	0.08	0.21^**^	0.28^**^	0.13^*^
9. Peer norms Alcohol (T1)									–	0.16^**^	0.18^**^	0.34^**^
10. Peer norms Cakes/Pastries (T2)										–	0.26^**^	0.13^*^
11. Peer norms Soda (T2)											–	0.22^**^
12. Peer norms Alcohol (T2)												–

* *p < 0.05*,

** *p < 0.01*.

### Predicting cakes/pastry consumption (Table 3a)^1^

All collinearity diagnostics were in the acceptable range (VIFs <1.85; Tolerance > 0.54; Durbin Watson = 2.10). The final regression model was significant [*F*_(9, 324)_ = 19.45, *p* < 0.001], and predicted 33% of variance in cake/pastry consumption at follow up. Gender (males = higher frequency), frequency of consumption at baseline, self-control, perceived peer norms at baseline, and change in perceived peer norms from baseline to follow up were all positive significant predictors of cake/pastry consumption at follow up. After controlling for other baseline variables (step 1 of the model) baseline perceived peer norms accounted for <1% of variance and was not a significant predictor at step 2. Changes in perceived peer norms and self-control were significant predictors and accounted for 3.3% of variance (step 3) in personal frequency consumption at follow up. The inclusion of self-control and changes in perceived peer norms at step 3 resulted in baseline perceived peer norms becoming a significant predictor in the model. There were no significant interactions between perceived peer norms and trait self-control (entered in step 4).

**Table 3:** **Multiple linear regressions investigating the longitudinal effect of perceived peer descriptive norms and self-control on frequency of unhealthy food/drinks consumption in university students**.

	**A. Cakes/pastries**		**B. Sugar containing sodas**		**C. Alcohol**	
	**B (*SE*)**	**95% CI**	**B (*SE*)**	**95% CI**	**B (*SE*)**	**95% CI**
Age	0.010 (0.014)	−0.016–0.037	−0.036 (0.019)	−0.073–0.002	−0.017 (0.007)^*^	−0.031–−0.003
Gender	−0.340 (0.160)^*^	−0.654–−0.026	−0.328 (0.231)	−0.783–0.127	−0.028 (0.085)	−0.195–0.138
BMI	0.012 (0.013)	−0.014–0.038	0.032 (0.019)	−0.005–0.069	−0.002 (0.007)	−0.016–0.011
Baseline Freq.	0.516 (0.050)^**^	0.418–0.613	0.636 (0.042)^**^	0.553–0.719	0.621 (0.044)^**^	0.534–0.708
Baseline Norm	0.377 (0.131)^**^	0.119–0.635	0.100 (0.207)	−0.306–0.506	0.127 (0.093)	−0.056–0.311
BSCS	−0.019 (0.008)^*^	−0.035–−0.002	−0.021 (0.012)	−0.045–0.003	0.001 (0.005)	−0.008–0.010
Norm change	0.387 (0.125)^**^	0.141–0.632	0.114 (0.155)	−0.191–0.418	0.111 (0.074)	–0.034–0.256
Baseline norm^*^BSCS	−0.025 (0.015)	−0.055−0.005	−0.012 (0.028)	−0.069−0.042	−0.011 (0.010)	−0.030–0.009
Norm change^*^BSCS	−0.028 (0.017)	−0.061–0.005	0.001 (0.020)	−0.040–0.040	−0.017 (0.009)	−0.036–0.001
	***R*^2^-Change**	***F*****-change**	***R*^2^-Change**	***F*****-change**	***R*^2^-Change**	***F*****-change**
Step 1	0.31	36.68^**^	0.46	69.09^**^	0.551	84.77^**^
Step 2	<0.01	1.99	<0.01	0.01	<0.01	0.21
Step3	0.03	8.28^**^	0.01	1.96	<0.01	1.00
Step 4	0.01	1.66	<0.01	0.12	0.01	1.75

* p < 0.05;

** *p < 0.01*.

B, unstandardised co-efficient; BMI, Body Mass Index; BSCS, Brief Self-Control Scale scores; Baseline Frequency, self-reported behavior at baseline; Baseline Norm, perceived peer norms at baseline. Norm Change, change in perceived peer norms between baseline and follow up; T1 Norm^*^BSCS, the interaction between perceived peer norms at baseline and self-control scores; Norm Change^*^BSCS, the interaction between change in perceived peer norms and self-control scores; Estimates from the final step of each regression (step four) are reported.

### Predicting sugar containing soda consumption (Table 3b)

All collinearity diagnostics were in the acceptable range (VIFs <1.40; Tolerance > 0.72, Durbin Watson = 1.84). The final regression model was significant [*F*_(9, 324)_ = 31.45, *p* < 0.001], and predicted 45% of variance in sugar containing soda consumption at follow up. The only significant predictor of personal frequency of sugar containing soda consumption at follow up was frequency of consuming sugar containing soda at baseline. No significant variance was predicted by perceived peer norms, trait self-control, or interactions between the two.

### Predicting alcohol consumption (Table 3c)

All collinearity diagnostics were in the acceptable range (VIFs <1.88; Tolerance > 0.53; Durbin Watson = 1.80). The final regression model was significant [*F*_(9, 334)_ = 38.39, *p* < 0.001], and predicted 50% of variance in alcohol consumption at time 2. Age and baseline frequency of alcohol consumption were significant predictors of personal frequency of alcohol consumption at follow up. No significant variance was explained by perceived peer norms, trait self-control, or interactions between the two.

### Attrition

To examine baseline differences between completers and non-completers we compared completers and non-completers on age, BMI, and self-control scores and found no significant differences (*ps* > 0.05). There was a significant observed difference between gender and attrition (*p* = 0.01), whereby there was a smaller percentage of females at time 2 (68.4%), compared to time 1 (76.2%).

In previous cross-sectional analyses (*n* = 1056), we found evidence (Robinson et al., 2014b, 2016) that perceived descriptive peer norms were significantly associated with personal consumption frequency for all three of the food/beverage types tested (cakes/pastries, sugar containing soda, alcohol), but here we found limited evidence for a longitudinal association between the two (*n* = 340). One potential explanation is that attrition from baseline to follow up was not random in nature and this may have resulted in us sampling participants in our longitudinal analyses who showed no or only a weak baseline cross-sectional association between perceived descriptive peer norms and consumption behavior. To address this, we tested whether the size of cross-sectional association between perceived descriptive peer norms and personal consumption frequency for the three food/beverage types was similar among participants we sampled at baseline and follow up (completers) vs. those who did not complete the follow up measures and therefore could not be included in the present analyses (non-completers). We did this by examining whether there were significant interactions between perceived peer norms and completer status in regression models that predicted personal consumption frequency at baseline for the three food/beverage types. We found no evidence (*ps* > 0.05) that the cross-sectional association between perceived descriptive peer norms and personal consumption frequency for any of the three food/beverage types differed between completers vs. non-completers. Thus, the lack of evidence for strong longitudinal associations between perceived descriptive peer norms and personal consumption frequency is unlikely to be explained by attrition resulting in us sampling participants in our longitudinal analyses who showed a weaker baseline association between these variables.

## General discussion

The aim of the present study was to examine whether perceived descriptive peer norms for different food/beverage types prospectively predict consumption of different food/beverages (cakes/pastries, sugar containing soda, and alcohol use) 1 year later. We found mixed evidence for the longitudinal association between perceived descriptive peer norms and personal frequency of consumption. For the food category cakes and pastries, in our final model, measures of both baseline and changes to perceived descriptive peer norms significantly predicted how frequently participants consumed cake/pastries 1 year later. However, baseline perceived descriptive peer norms accounted for only a very small amount of variance of future consumption of cakes/pastries (<1%) and the statistical significance of the association between baseline perceived descriptive peer norms and consumption of cakes/pastries was dependent on which other variables analyses were adjusted for. Changes in perceived descriptive peer norms had a small predictive effect on future consumption of cakes/pastries, but this finding may be explained by reverse causality; if a person has increased their consumption of cakes/pastries, they may be more likely to believe others have too. We found no evidence that baseline perceived descriptive peer norms or changes to peer norms prospectively predicted how frequently participants consumed sugar containing soda or six or more alcoholic drinks in one occasion at follow-up. Moreover, because self-control may be required to inhibit the influence of perceived descriptive peer norms on behavior (Burkley et al., 2011; Salmon et al., 2014), we hypothesized that any longitudinal effect of perceived descriptive peer norms may be particularly pronounced among participants with low levels of trait self-control. However, we found no evidence in support of this relationship.

The present results therefore provide mixed evidence in support of our hypotheses. Based on baseline data from this cohort study (Robinson et al., 2014b, 2016) and a number of studies showing a cross-sectional association between perceived descriptive peer norms and consumption behavior (e.g., Ball et al., 2010), our main hypotheses was that perceived descriptive peer norms would prospectively predict consumption behavior. We did find that changes to consumption behavior over time occurred, whereby significant proportions of participants at follow up were eating and drinking the studied foods and beverages more or less frequently than they were at baseline. However, perceived descriptive norms were not a reliable predictor of future behavior in the present study and this is consistent with a previous smaller scale prospective study (Louis et al., 2007). If the present results are replicated, then reconciling different results between cross-sectional vs. longitudinal studies will be of importance. It may be the case that the previously identified association between perceived descriptive peer norms and consumer behavior (Louis et al., 2007; Ball et al., 2010; Lally et al., 2011; Robinson et al., 2014b, 2016; Staunton et al., 2014; Pelletier et al., 2016) is largely the result of reverse causality, as opposed to descriptive norms shaping everyday consumer behavior.

A limitation of the present work was that attrition from baseline to follow up was relatively high. Although we observed a high attrition rate, the lack of significant longitudinal associations observed do not appear to be due to insufficient statistical power, as for both sugar containing soda and alcohol consumption, perceived descriptive peer norms accounted for close to 0% variance of personal consumption behavior at follow up in our analyses. We found no evidence of differences between completers and non-completers in terms of the cross-sectional relationship between perceived descriptive peer norms and consumption behavior in additional analyses. Moreover, the characteristics of the participant sampled at follow-up vs. those who were lost to attrition were not markedly different. Finally, estimates of bias due to random data loss in cohort studies tend to be low (Deeg, 2002; Kristman et al., 2004), again indicating that the failure to identify consistent longitudinal effects of perceived descriptive peer norms on future consumption in the present study is unlikely to have been due to attrition.

Why we previously observed cross-sectional associations between perceived descriptive peer norms for all food/beverage types (Robinson et al., 2014b, 2016) but only a longitudinal effect on cakes/pastries (but not sugar containing soda or alcohol) in the present study warrants attention, as we did not have a-priori hypotheses concerning potential differences between food types in the present study. Concerning the potential influence of trait self-control, we found that lower levels of self-control predicted increased consumption of cakes/pastries over time, but did not predict changes to consumption of sugar containing soda or alcohol. It may be the case that cakes/pastries are perceived by consumers as being particularly indulgent or tempting and inhibiting temptation is particularly difficult when consumption of such foods is perceived to be normative (Robinson et al., 2016). However, this interpretation is speculative. Furthermore, if this were the case then we may also expect that the longitudinal effect of perceived descriptive peer norms to be particularly pronounced among individuals with low self-control, but this was not the case in the present study.

Here we focused on perceived descriptive peer norms, as the majority of evidence linking normative influence and eating behavior has focused on descriptive norms (Robinson et al., 2014c; Robinson, 2015). However, it is plausible that other types of peer norm, such as perceptions of social support or what others approve of (Povey et al., 2000; Stok et al., 2014a) could predict longitudinal changes in behavior. Thus, future work would benefit from examining the longitudinal effect of other forms of social norms. In the present study, the norm referent group referred to the eating/drinking behavior of “other students.” Although students are likely to psychologically identify with other students, it may be the case that a more proximal social reference group would elicit a greater influence on future behavior (Stok et al., 2014b, 2016). For example, it may be the case that students are most influenced by their perception of how frequently other students from their own university eat/drink different food/beverage types. Further. examination of whether different norm referent groups affect longitudinal influences on eating or drinking behavior would now be informative.

Although this is one of the first longitudinal examinations of the association between perceived descriptive peer norms and personal food/drink consumption, the present study had limitations. First, we were only able to examine a limited number of food/beverage types and it would be more informative to examine the longitudinal association between perceived descriptive peer norms and a broader range of food/beverage types. The majority of research into the experimental effects of descriptive peer norms on consumption behavior has focused on a narrow demographic (university students), so here we sampled a similar population and this means the generalizability of our findings to other populations is limited. Moreover, we relied on single item self-report measures of personal consumption frequency and perceived descriptive peer norms. Although these measures were based on similar recent studies which have shown convincing evidence of a cross-sectional association between perceived descriptive peer norms and personal consumption behavior (Ball et al., 2010; Robinson et al., 2014b, 2016), future work could make use of more objective measures. Our main focus was also on frequency of consumption (i.e., how often a food or beverage type is consumed), so work examining the potential influence of perceived descriptive peer norms on portion size selection and/or total energy intake would also be valuable. Finally, we examined the prospective effect of perceived descriptive peer norms on behavior 1 year later and it may be the case that perceived descriptive peer norms would be more likely to predict future behavior over a shorter follow up period. Given these caveats the present study should act as an initial examination of the longitudinal effect of perceived descriptive peer norms on food/drink consumption. Further, work is now needed to address the limitations of the present work and examine the replicability of our findings.

## Conclusions

In the present longitudinal study of young adults, beliefs about how often one's peers eat or drink specific food and beverages types had limited effect on future eating and drinking behavior.

## Author contributions

AJ and ER conceived the study design. AJ performed statistical analyses. Both AJ and ER drafted the manuscript and approved the final version.

## Funding

ER was supported by an MRC New Investigator Research Grant (MR/N000218/1).

### Conflict of interest statement

The authors declare that the research was conducted in the absence of any commercial or financial relationships that could be construed as a potential conflict of interest.

## Footnotes

^1^Analyzing the data using ordinal regression models produced the same results as reported here. linear models are reported for ease of interpretation.
